# Correlations between cytomegalovirus, Epstein-Barr virus, anti-ganglioside antibodies, electrodiagnostic findings and functional status in Guillain-Barré syndrome

**Published:** 2014

**Authors:** Aliakbar Taheraghdam, Peyman Pourkhanjar, Mahnaz Talebi, Mohammadreza Bonyadi, Ali Pashapour, Ehsan Sharifipour, Reza Rikhtegar

**Affiliations:** 1 Department of Neurology, Neurosciences Research Center (NSRC), School of Medicine, Tabriz University of Medical Sciences, Tabriz, Iran; 2 Neurologist, Department of Neurology, Neurosciences Research Center (NSRC), School of Medicine, Tabriz University of Medical Sciences, Tabriz, Iran; 3 Department of Neurology, Neurosciences Research Center (NSRC), Student Research Committee, School of Medicine, Tabriz University of Medical Sciences, Imam Reza Hospital, Tabriz, Iran

**Keywords:** Guillain-Barré Syndrome, Anti-Ganglioside Antibodies, Cytomegalovirus, Epstein-Barr Virus, Electrodiagnostic Findings

## Abstract

**Background:**

Due to underlying autoimmune background of Guillain-Barré syndrome (GBS), the possible role of infectious agents cytomegalovirus (CMV) and Epstein-Barr virus (EBV) and also due to association of anti-ganglioside antibodies with GBS, the present study aimed to investigate the associations between serum anti-ganglioside antibodies (AGA) level, type of infection and electrodiagnostic (ED) findings with the severity and three-month functional outcome of patients with GBS.

**Methods:**

In a prospective study, 30 patients with GBS were selected and before starting the treatment, baseline serum samples of patients were obtained for measuring the serum AGA including the antibodies against GQ1b, GT1b, GD1a, GD1b, GM1, GM2, GM3 and strains of CMV and EBV. All the patients were precisely examined for ED findings. Functional status of patients on admission and three months after admission were recorded according to the modified Rankin scale (mRS).

**Results:**

The results of patients’ serum assessment revealed that CMV IgM was positive in one patient (3.3%), CMV IgG in 29 patients (96.7%) and EBV IgG in 27 patients (90%). Anti-GM1 was found in 3 patients (10%) and anti-GM3 was found only in one patient (3.3%). However, no statistical significant association was found between the AGA and strain of the disease and ED findings.

**Conclusion:**

Despite the coexistence of AGA and serum antibodies against CMV and EBV in some GBS patients, there was not clear association in this regard. However, the AGA was positive in patients who suffered from severe phase of the disease.

## Introduction

Guillain-Barre syndrome (GBS) is an autoimmune disorder characterized by a group of heterogeneous and different clinical, electrophysiological, and pathological findings and subtypes.^1, 2^ The incidence of this acute neuropathy is common, with an annual incidence of 1.1 to 1.8 in each 100000 persons.^3^ In this syndrome, it is believed that various infections stimulate immune system leading to a cross-reaction with nervous system (against antigens of peripheral neurons) and thereby demyelination of neurons and, eventually, initiation of nervous signs and symptoms.^4–6^ It has been shown that the most common preceding infection leading to this disease is Campylobacter jejuni enteritis and other leading infections are Cytomegalovirus (CMV), Epstein-Barr Virus (EBV), Mycoplasma pneumonia and Haemophilus influenzae.^7^ This syndrome is often diagnosed by clinical findings, but electrophysiological findings are also helpful which determine the important subtypes of this syndrome: acute inflammatory demyelinating polyneuropathy (AIPD), acute motor axonal neuropathy (AMAN), acute motor and sensory axonal neuropathy (AMSAM) and Miller Fisher syndrome (MFS) in which the cranial nerves are involved.^8, 9^ Gangliosides are glycosphyngolipid components located in higher density in peripheral nervous system, especially in axons of the neurons.^10, 11^ The humoral reactions against these glycolipids play the crucial role in the pathogenesis of GBS.^12, 13^ Antibodies against different complexes of gangliosides include GM1, GM2, GD1a, GD3, GT1a, and GQ1b all are detectable in GBS patients.^14, 15^ These anti-ganglioside antibodies (AGA) in different preceding infections are accompanied with different clinical demonstrations.^16^ Accompanying of GBS with CMV infection and antibody anti-GM2 has been frequently reported.^17, 18^ In addition, some studies have reported the correlation between some AGA with GBS disease and electrodiagnostic (ED) alterations.^1^ Due to geographical-regional differences in common patterns of GBS and common infections and also due to regional variability of causative gangliosides in each strain, in this study we have measured the levels of anti-ganglioside IgG antibodies in the serum of adults with GBS and then evaluated this correlations with recent diagnosed infections and different ED patterns and functional outcome of this patients.

## Materials and Methods

In this prospective and descriptive-analytical study, 30 adult patients with GBS who were admitted from August 2011 to August 2012 to the Neurology ward of Imam Reza hospital in Tabriz (northwest of Iran) were studied. The inclusion criteria were: 1) symptoms and signs compatible with GBS, 2) initiation of the symptoms in interval less than one week before blood collection, 3) ages 18 to 80 years old and 4) informed consent of patients for participation in this study. If patients have one of the following findings, they were excluded from the study: 1) failure in confirmation of GBS with electrophysiological assessment, 2) previous history of any disabling disease, and 3) unwillingness of patients to continuing the study.

To identify patients with Guillain-Barre syndrome, first all patients in the acute phase of polyneuropathy disease, complete systemic and neurological examinations were performed by an expert neurologist and fulfilled the Asbury et al criteria for GBS.^19^ Then the standard ED study was performed by an experienced neurologist. The ED testing and diagnosis of GBS was done based on Meulstee et al criteria.^20^ For all patients, the clinical information including demographic information, history of recent gastrointestinal or respiratory infection in the last month was taken. Furthermore, the type of syndrome has been determined using clinical and paraclinical investigations according to the ED findings. Prior to treatment, 10 ml non-fasting blood samples were obtained in a tube containing citrate from all patients, within the first week from onset of symptoms. Then the plasma was obtained after 15 minute centrifugation (3000 rpm) at room temperature and transferred to refrigerator with a temperature of -80° C. The levels of anti-gangliosides antibodies including antibodies against GQ1b, GT1b, GD1a, GD1b, GM1, GM2, and GM3 were measured using commercial Euroimmune kits by enzyme-linked immunosorbent assay (ELISA) method. In addition, the level of antibodies to CMV and EBV was also assessed using this method. Once admitted in the ward, patients were examined by an expert neurologist and the severity of disability was determined and recorded according to motor function and modified Rankin scale (mRS). Furthermore, after three months, patients were re-examined by the same neurologist and the degree of disability were recorded. This study was approved by the ethics committee of Tabriz University of Medical Sciences, which was in agreement with the Declaration of Helsinki and written consent was obtained from all patients prior of the study. None patient was imposed by any additional costs.

### Statistical analysis

All data in this study were analyzed using statistical software SPSS16. To compare the qualitative findings, chi-square test was used. The comparison and analysis of quantitative findings between groups, was made using Independent t test. For comparing of qualitative data between two groups, Fisher's exact test and for quantitative data between two groups, the nonparametric Mann-Whitney U test was performed. In addition, to evaluate the relationship (correlation) between anti-ganglioside and other variables, Pearson's correlation test was used. A P value less than 0.05 was considered statistically significant.

## Results

**Figure 1:**
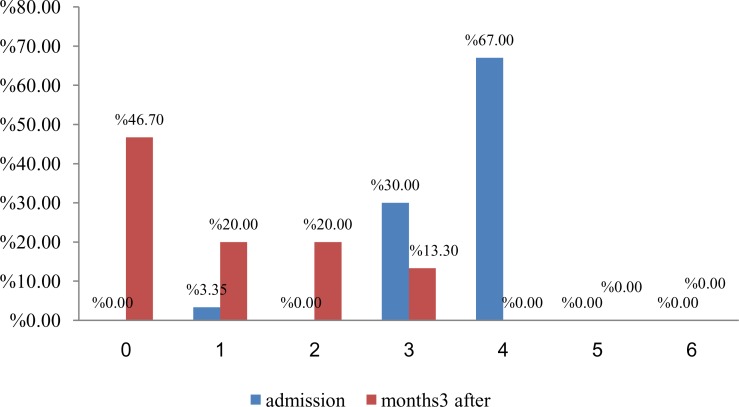
Grading of patients disability during hospitalization and three months later, according to modified Rankin scale (mRS).

Among 30 patients with an average age of 50.33±17.66 (20-83) years old, 21(70%) of them were males and 9 (30%) were females. Twenty five patients (83.3%) had the history of recent underlying infection (preceding infectious context) including respiratory in 18 (60%) or gastrointestinal symptoms in 7 (23.3%). The most common pattern of involvement of patients based on ED findings was AIDP in 14 (46.6%) patients and the other ED patterns were: AMAN in 7 (23.3%), AMSAN in 8 (26.7%) and MFS in 1 (3.3%) patients. The average mRS of patient during hospitalization time was 3.6(1-4), Figure 1 shows the degree of disability of patients on the basis of mRS, during admission and three months after treatment. It would be mentioned that 6.6% patients were required mechanical ventilation. As it shown, in the assessing the serum levels of infectious factors in sera of patients it was observed that CMV-IgM was positive only in serum of one patient (3.3%) but CMV-IgG were positive in 29 patients (96.7%), EBV-IgG in 27 patients (90%) and nobody with EBV-IgM. In addition, serum AGA was found only in 4 patients: Anti GM1 in 3 (10%) and Anti GM3 in 1(3.3%) patients. Although all AGA was found in male patients, there wasn't significant relationship between AGA and sex. Seventy five percent of positive cases of AGA were in patients with no history of respiratory or gastrointestinal infections or symptoms. Table 1 comparise the different findings between cases with and without AGA. As it can be observed, there was a significant negative correlation between the level of AGA and respiratory disease (p = 0.007 and r = -0.48) and there was a significant positive correlation between the level of AGA and CMV-IgM (P = 0.008 and r = 0.47). In the remaining cases, there was no statistically significant correlation. In addition, 75% of positive cases of AGA were disable and had mRS = 4, and remaining had mRS = 3. AGA were found in four patients as follows: 1) a 54 years old man with AIDP which his EBV-IgG and CMV-IgG antibodies were positive and the others were negative, and his AGA was Anti GM1, 2) A 58 years old man with AMAN, which his CMV-IgG antibody was positive and the others were negative, and his AGA was Anti GM1, 3) a 25 years old man with AMAN, which his EBV-IgG, CMV-IgG and CMV-IgM antibodies were positive and the others were negative, and his AGA was Anti GM1, 4) a 60 years old man with AMSAN, which his EBV-IgG and CMV-IgG antibodies were positive and the others were negative and his AGA was Anti GM3. mRS during the hospitalization time for the first patient was 3 and for the remaining patients was 4. On the other hand, the three months mRS of all patients was 1, except patients no.2 that was 3. Furthermore, patient 2 and 4 were required mechanical ventilation. It should be also noted that there was no significant correlation between these antibodies and GBS patterns. The functional status of all patients after three months in comparison with on admission mRS showed a significant improvement (P < 0.001). Figure 1 demonstrates grading of patients disability during hospitalization and three months later, according to mRS. The distribution of ED patterns of GBS in EBV-IgG positive and CMV-IgG positive patients have been shown in Table 2. Significant correlation between the severity of disability (mRS) during admission or after 3 months and microbial strains (CMV-IgM, CMV-IgG, EBV-IgM and EBV-IgG) was not found. In addition, there wasn't any significant relationship between microbial strains (CMV-IgM, CMV-IgG, EBV-IgM and EBV-IgG) and ED findings.

**Table 1:** Comparison of multiple variables between caces of with or without anti-ganglioside antibody

	Negative anti-ganglioside	Positive anti-ganglioside	P
Age	49.25 ± 16.35*	50.50 ±18.15*	0.93
Sex (male)	4 (100%)	17 (65.4%)	0.28
Respiratory disease	0	18 (69.2%)	0.01
Gastrointestinal disease	1 (25%)	6 (23.1%)	0.93
IgM.CMV	1 (25%)	0	0.13
IgG.CMV	4 (100%)	25 (96.2%)	0.9
IgG.EBV	3 (75%)	24 (92.3%)	0.36

* Values are presented as mean ± SD or n (%). CMV: Cytomegalovirus; EBV: Epstein-Barr virus.

**Table 2:** Comparison of IgG between multiple patterns and GBS subtypes

Electrodiagnostic patterns	CMV IgG+ (%)	EBV IgG+ (%)
AIDP	14 (48.3)	13 (48.1)
AMSAN	8 (27.6)	8 (29.6)
AMAN	6 (20.7)	5 (18.5)
MFS	1 (3.4)	1 (3.7)

CMV: Cytomegalo virus; EBV: Epstein-Barr virus; AIDP: Acute inflammatory demyelinating polyneuropathy; AMAN: Acute motor axonal neuropathy; AMSAN: Acute motor and sensory axonal neuropathy; MFS: Miller fisher syndrome.

## Discussion

The results of current investigation showed that despite positive antibody titer against CMV and EBV, AGA were positive only in a small percentage of patients (4 patients) which they were of Anti-GM1 and Anti-GM3 subtypes. Anti GM1 were found in 2 different ED patterns, one in AMAN and another in AIDP, and Anti-GM3 were found in a patient with AMSAN. The AGA was found in patients who had a worse initial functional status but after three months, most of them had been improved except one with residual disability. CMV infection is the second most common antecedent infection before GBS and there are evidences of pathogenicity of CMV infection in 5-22% of this syndrome in several studies.^5^ Anti-GM2 often express in GBS following infection with CMV.^16^ However, in our study 75% of patients with positive AGA and anti-CMV antibody had Anti-GM1 and the remaining had anti-GM3. In a study conducted with Hao and colleagues, AGA was found in 14% of patients.^21^ Similarly in the present study we found these antibodies in 13.3% of patients with GBS. Jacobs et al in their study found Anti-GM1 in 22 patients (18%) and Anti-GQ1 in 5 patients (4%).^1^ In the present study, 3 patients (10%) had Anti-GM1 and only one patient (3.3%) had Anti-GM3. Kornberg et al reported that there is a strong and significant correlation between Anti-GM1 antibodies and AMAN subtype.^22^ On the other hand, Yuki and his colleagues demonstrated that Anti-GD1a antibodies also could be found in AMAN patients.^23^ In another study conducted by Ogawara et al, it was shown that the most common AGA were anti-GM1, anti-GD1a and anti-Ga1NAc-GD1a, which all of them had significant relationship with AMAN subtype.^24^ In the current study, we found that AGA in AMAN and AIDP was Anti-GM1, and AGA in the ED pattern of AMSAN was Anti-GM3, but there was no possibility to analyze the relationship between AGA and subtypes of GBS. Yuki et al reported the subtype of AMAN in association with Campylobacter jejuni enteritis;^23^ while the results of this study revealed that most of patients with positive CMV-IgG and EBV-IgG had AIDP subtype of GBS. In addition, AGA usually was found in serum of patients who were positive for CMV-IgG and EBV-IgG. In Sivadon et al observation on 264 patients with GBS, they found that primary or recent infection with CMV was associated with disease in 15% of patients, which most of them were complicated with upper respiratory infection and severe form of the disease (37.5%), and Anti-GM2 was the AGA in their serum.^25^ Similarly, in an 8-year survey on 249 patients with GBS conducted by Caudie and colleagues, in 36% of patients AGA were diagnosed using immunodot-blot method; among them, in 11 (8%) patients with positive AGA who also had severe form of the GBS there was a recent infection with CMV. The type of positive AGA in these 11 patients was anti-GM2 IgM.^26^ Khalili-Shirazi et al in a study have evaluated the AGA in the serum of different groups of patients (GBS with recent CMV infection, GBS without CMV infection, with other neurological diseases, with a recent CMV infection but without neurological involvement, with recent EBV infection but without neurological involvement) and 20 normal control subjects by the means of ELISA method and the following results were obtained: IgM antibodies were found to gangliosides GM2 (six of 14 patients), GM1 (four of 14), GD1a (three of 14) and GD1b (two of 14) in the serum samples of GBS patients with CMV infection, but not in GBS patients without CMV infection. IgM antibodies were also found to gangliosides GM1, GD1a, and GD1b in one of 11 patients with other neurological diseases, to ganglioside GM1 in one of 11 non-neurological CMV patients, and to ganglioside GD1b in one of 20 normal control subjects. Some patients with EBV infection had IgM antibodies to gangliosides GM1 (five of 11), GM2 (three of 11), and GD1a (two of 11).^18^ Nafissi et al in an investigation on Thirty five patients with GBS and 35 matched control population in the same period of time in Iran, found IgM antibody against CMV in 6 cases and 2 controls, IgG anti-Haemophilus influenzae (HI) in 34 patients and 31 controls andIgM antibody against EBV infection in one patient. They concluded that despite the more frequency of serologic evidences of these evaluated infections in GBS patients, only HI infection (with high titer of IgG antibody) significantly related to GBS.^27^

In the current study, the most of AGA-positive patients had Anti-GM1 (75%) and the rest (25%) had Anti-GM3. In addition, Anti-GM2 was not positive in none of them and all of them had severe forms of disease. In the serum assessment of these patients to determine the relationship with infectious agents, it was shown that Anti-CMV IgG was positive in all patients, Anti-EBV IgG was positive in 75% and Anti-CMV IgM was found only in 25% (who had recent infection with the CMV and his positive AGA was Anti-GM1). Furthermore, in determining the association of AGA with ED patterns of GBS, it should be mentioned that Anti-GM1 was associated with AIDP and AMAN and Anti-GM3 was associated with AMSAN form.

## Conclusion

In spite of coexistence of AGA and serum antibodies against CMV and EBV, there was no clear and significant correlation in this regard. However, the end result of this study was that if the disability of patients is higher, the possibility of positive AGA is higher. It was also revealed that Anti-GM1 and Anti GM3 may be associated with CMV and EBV. However, comprehensive studies in the future can further highlight the degree of relationship and involvment of CMV and EBV infection and production of AGA in GBS.
